# Chitinase mRNA Levels Determined by QPCR in Crab-Eating Monkey (*Macaca fascicularis*) Tissues: Species-Specific Expression of Acidic Mammalian Chitinase and Chitotriosidase

**DOI:** 10.3390/genes9050244

**Published:** 2018-05-09

**Authors:** Maiko Uehara, Eri Tabata, Kazuhiro Ishii, Akira Sawa, Misa Ohno, Masayoshi Sakaguchi, Vaclav Matoska, Peter O. Bauer, Fumitaka Oyama

**Affiliations:** 1 Department of Chemistry and Life Science, Kogakuin University, Hachioji, Tokyo 192-0015, Japan; bm17005@ns.kogakuin.ac.jp (M.U.); tbt16024@yahoo.co.jp (E.T.); horn7mi.brass@gmail.com (M.O.); bt11532@ns.kogakuin.ac.jp (M.S.); 2 Japan Society for the Promotion of Science (DC1), Koujimachi, Chiyoda-ku, Tokyo 102-0083, Japan; 3 Department of Psychiatry and Behavioral Sciences, Johns Hopkins University School of Medicine, 600 North Wolfe Street, Meyer 3-166A, Baltimore, MD 21287, USA; kishii2@jhmi.edu (K.I.); asawa1@jhmi.edu (A.S.); 4 Department of Clinical Biochemistry, Hematology and Immunology, Homolka Hospital, 150 00 Prague, Czech Republic; vaclav.matoska@homolka.cz (V.M.); peter.bauer@bioinova.cz (P.O.B.); 5 Bioinova Ltd., 142 20 Prague, Czech Republic

**Keywords:** acidic mammalian chitinase, asthma, chitinolytic activity, chitotriosidase, crab-eating monkey, human, mouse, nonhuman primate model, qPCR, species-specific gene expression

## Abstract

Mice and humans express two active chitinases: acidic mammalian chitinase (AMCase) and chitotriosidase (CHIT1). Both chitinases are thought to play important roles in specific pathophysiological conditions. The crab-eating monkey (*Macaca fascicularis*) is one of the most frequently used nonhuman primate models in basic and applied biomedical research. Here, we performed gene expression analysis of two chitinases in normal crab-eating monkey tissues by way of quantitative real-time polymerase chain reaction (qPCR) using a single standard DNA molecule. Levels of AMCase and CHIT1 messenger RNAs (mRNAs) were highest in the stomach and the lung, respectively, when compared to other tissues. Comparative gene expression analysis of mouse, monkey, and human using monkey–mouse–human hybrid standard DNA showed that the AMCase mRNA levels were exceptionally high in mouse and monkey stomachs while very low in the human stomach. As for the CHIT1 mRNA, we detected higher levels in the monkey lung when compared with those of mouse and human. The differences of mRNA expression between the species in the stomach tissues were basically reflecting the levels of the chitinolytic activities. These results indicate that gene expression of AMCase and CHIT1 differs between mammalian species and requiring special attention in handling data in chitinase-related studies in particular organisms.

## 1. Introduction

Chitin, a polymer of *N*-acetyl-d-glucosamine, is the second most abundant polysaccharide in nature [1,2]. It is an integral component of the exoskeletons of crustaceans and insects, the microfilarial sheaths of parasitic nematodes, and fungal cell walls [1,2,3].

Chitinases are enzymes that digest the chitin polymer. Although mammals do not produce chitin, mice and humans have two genes that encode active chitinases—chitotriosidase (CHIT1) and acidic mammalian chitinase (AMCase) [2,3,4]. CHIT1 was the first mammalian chitinase to be purified and cloned [5,6,7]. Later, AMCase was identified as a compensatory enzyme for CHIT1 and named for its acidic isoelectric point [8,9].

Previous studies have shown associations between the expression of the mammalian chitinases and specific pathophysiological conditions. For instance, the levels of CHIT1 are elevated in Gaucher disease, smokers and chronic obstructive pulmonary disease (COPD), and in the cerebrospinal fluid of patients with Alzheimer’s disease [5,10,11,12,13,14,15]. CHIT1 is expressed in the eye and lacrimal gland and targets a microbial spectrum different from lysozyme [16].

AMCase expression is upregulated during allergic airway responses in mouse models of asthma [17]. Polymeric chitin induces AMCase expression and the recruitment of immune cells that are associated with allergy and asthma [18]. In addition, several genetic variants of AMCase are associated with bronchial asthma in humans [19,20,21,22]. Recent studies using AMCase-deficient mice have shown that AMCase is a constitutively produced enzyme necessary for chitin degradation in the airways to maintain lung function [23] and that it plays a critical role in the protective immune response to gastrointestinal nematodes in the host gastrointestinal tract [24].

Previously, we established a quantitative reverse transcriptase-coupled PCR (qPCR) system using a single standard DNA molecule which allows us to characterize different chitinases expression profiles in detail [25,26,27]. More recently, we have shown that mouse AMCase and chicken Chia, a homologue of AMCase, as well as pig AMCase can function as protease-resistant major glycosidases in the respective digestive systems [27,28,29]. We have found that not all animals express AMCase in stomach tissues at high levels. Specifically, herbivorous and carnivorous animals such as bovine and dog have very low capability to digest chitin when compared to omnivorous animals [30].

Monkeys are genetically and physiologically similar to humans. They are frequently used in the drug discovery process preceding clinical trials to confirm drug safety and efficiency. The crab-eating monkey (*Macaca fascicularis*) is one of the most important nonhuman primate animal models for biomedical research [31] while belonging to the primates most tolerant of habitat disturbance by humans [32]. For example, the monkeys develop naturally occurring osteoarthritis (OA) that closely resembles the human disease. Since the chitinase-like protein YKL-40 is associated with OA, the monkeys represent a particularly useful animal model for studies on YKL-40 [33].

In accordance to the term “crab-eating monkey”, these primates feed on crabs but also on other chitin-containing organisms such as crustaceans and insects. The lack of genetic and biochemical information on this species has represented a significant obstacle for its broader use [31,34]. Clarifying biochemical information should further enhance use of the crab-eating monkey as a nonhuman primate animal model. Krykbaev et al. reported that macaca acidic chitinase (MACase) is highly expressed in the stomach and that the DNA sequence of the monkey AMCase has a higher identity percentage to the human pseudogene CHIAP2 than to human AMCase [34].

Here, we aimed to investigate three main questions: (i) is the monkey similar to human regarding the chitinases mRNA and protein levels; (ii) does AMCase function as a chitin-digestive enzyme in the monkey stomach; (iii) are the chitinase levels high in the lung tissues to maintain the airway function (crab-eating monkeys live in wetland, an environment with substantial amounts of airborne fungi)? To answer these questions, we performed gene expression analysis of chitinases in normal crab-eating monkey tissues by qPCR [25,26,27,28,29,30]. We also carried out comparative gene expression analysis using monkey–mouse–human hybrid standard DNA enabling a direct cross-species comparison of these genes’ expression between mouse, monkey, and human [26]. We report that the expression pattern of AMCase in monkey is similar to mouse in digestive and respiratory systems, whereas the expression pattern of monkey CHIT1 in lung was different from those in mouse and human. In addition, these mRNA differences among mouse, crab-eating monkey, and human stomach tissues were essentially reflecting differences in the chitinolytic activities. Thus, gene expression patterns of chitinases are species specific.

## 2. Materials and Methods

### 2.1. RNA and Complementary DNA Preparation

The qPCR system has been designed according to the Minimum Information for Publication of Quantitative Real-Time PCR Experiments (MIQE) guidelines [35,36]. The guidelines evaluate qPCR experiments to help increase experimental transparency.

Monkey Total RNA Panel (Funakoshi Co., Ltd., Tokyo, Japan), Human Total RNA Panel (Takara Bio USA, Inc., Mountain View, CA, USA), and Mouse Total RNA Panel (Takara Bio USA, Inc.) were used to examine the distribution of chitinase transcripts in normal crab-eating monkey, human, and mouse tissues. In addition, total RNA was reverse-transcribed into complementary DNA (cDNA) essentially as described previously [25,26,27].

### 2.2. Selection of Primer Pairs for qPCR

Primers for qPCR were designed using PrimerQuest Input (Integrated DNA Technologies, Coralville, IN, USA) and their suitability was evaluated based on the presence of single products, as reflected by a single melting temperature (Tm) [25,26,27]. The primers’ sequences are listed in Supplementary Table S1.

### 2.3. Construction of the Monkey Standard DNA and qPCR

**Figure 1:**
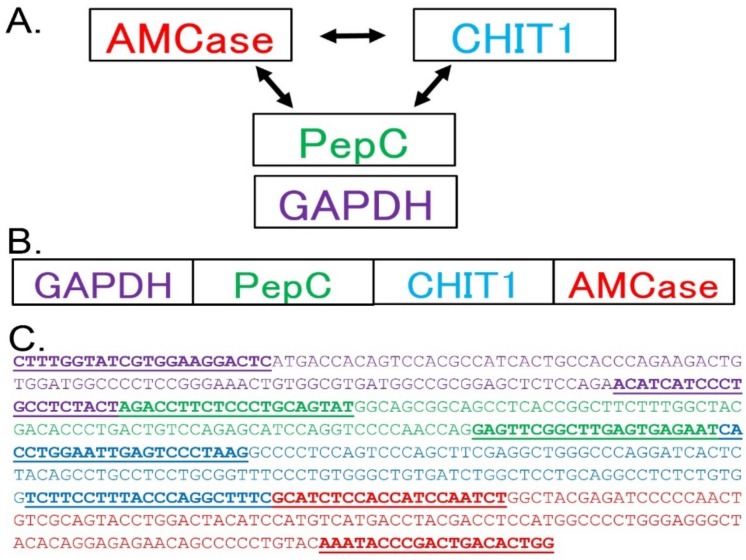
Strategy for the comparison of the gene expression levels of four crab-eating monkey genes. (**A**) We compared the expression levels of the acidic mammalian chitinase *(AMCase*) and chitotriosidase (*CHIT1*) genes. To evaluate chitinase levels in various monkey tissues, we used *glyceraldehyde-3-phosphate dehydrogenase (GAPDH)* and *pepsinogen C* (*Pep C*) as reference genes (*Pep C* only for stomach) in the same scale by using our quantitative reverse transcriptase-coupled PCR (qPCR) system. (**B**) Schematic representation of the standard DNA used for the qPCR. The target fragments for *GAPDH*, *pepsinogen C*, *CHIT1*, and *AMCase* complementaries DNA (cDNAs) were combined at a one-to-one ratio in a single DNA fragment. (**C**) Nucleotide sequence of the standard DNA containing four cDNA fragments (shown in different colors) covering the PCR target regions.

In a previous study using crab-eating monkey, Krykbaev et al. reported on MACase [34]. MACase is registered as AMCase (accession number NM_001284548.1) in the National Center for Biotechnology Information (NCBI) GenBank. Coding sequences of AMCase (GenBank accession number NM_001284548.1, nucleotides 560–707 of the AMCase cDNA), CHIT1 (GenBank accession number XM_005540484.1, nucleotides 1479–1626), pepsinogen C (GenBank accession number XM_005553043.1, nucleotides 455–567), and glyceraldehyde-3-phosphate dehydrogenase (GAPDH) (GenBank accession number NM_001319428.1, nucleotides 502–634) DNA containing regions amplified by the selected primers (described above) were synthesized and inserted into pTAKN-2 vector by Eurofins Genomics (Tokyo, Japan). The standard DNA (542 bases; see Figure 1B,C) was prepared by PCR reamplification from the plasmid DNA using the forward primer 5′-CTTTGGTATCGTGGAAGGACTC-3′ and the reverse primer 5′-CCAGTGTCAGTCGGGTATTT-3′. qPCR was essentially performed as described previously [25,26,27]. 2.4. Construction of the Monkey–Mouse–Human Hybrid Standard DNA and Qpcr.

The hybrid standard DNA (1086 bases; see Figure 4A,B) used for the quantification of the transcript levels by real-time PCR was constructed as follows: coding sequences of monkey genes (stated above), mouse *AMCase* (GenBank accession number NM_023186.3, nucleotides 1333–1413 of the mouse *AMCase* cDNA), mouse *Chit1* (GenBank accession number NM_001284525.1, nucleotides 1402–1470), mouse *pepsinogen C* (GenBank accession number NM_025973.3, nucleotides 887–968), mouse *GAPDH* (GenBank accession number XM_001476707.5, nucleotides 766–842), human *AMCase* (GenBank accession number NM_201653.3, nucleotides 1300–1361), human *CHIT1* (GenBank accession number NM_003465.2, nucleotides 454–508), human *pepsinogen C* (GenBank accession number NM_002630.3, nucleotides 1049–1109), and human *GAPDH* (GenBank accession number NM_001289746.1, nucleotides 383–439) DNA containing regions amplified by the selected primers (described above) were synthesized and inserted into pTAKN-2 vector by Eurofins Genomics. The hybrid standard DNA was amplified using the forward primer 5′-CTTTGGTATCGTGGAAGGACTC-3′ and the reverse primer 5′-AGCTGGAGCCGTGCAACTT-3′ and was thereafter used as the standard DNA for qPCR.

### 2.4. Mouse, Monkey, and Human Stomach and Lung Extracts

Total protein extracts from normal mouse, crab-eating monkey, and human stomach or lung tissues were commercially available (protein concentration 5 mg/mL) (Zyagen, San Diego, CA, USA).

### 2.5. Chitinase Enzymatic Assays

Chitinolytic activity was determined using a synthetic fluorogenic substrate, 4-methyl umbelliferyl β-d-*N, N*′-diacetyl chitobioside (Sigma-Aldrich, St. Louis, MO, USA) in McIlvaine’s buffer (0.1 M citric acid and 0.2 M Na_2_HPO_4_; pH 2.0 or pH 5.0) at 37 °C for 30 min as described previously [22,26]. The fluorescence of liberated 4-methyl umbelliferon was measured by GloMax Discover Multimode Microplate Reader (Promega, Madison, WI, USA) with excitation at 365 nm and emission at 445 nm.

### 2.6. Statistical Analysis

Biochemical data were compared using Student’s *t*-test.

## 3. Results

### 3.1. Characterization of the qPCR System for Detection of Chitinases and Reference Genes in Crab-Eating Monkey Tissues

Quantification of AMCase and CHIT1 mRNA levels is a key step toward understanding of the in vivo regulation of chitinases in the crab-eating monkey. In this study, we aimed to compare the gene expression levels of the *AMCase* and *CHIT1* genes in normal crab-eating monkey tissues (Figure 1A). Previously, we established a qPCR system that can determine the mRNA levels of the two mammalian chitinases in mouse and human tissues and compare their levels with those of reference genes using a single scale [25,26,27]. We used *GAPDH* as the reference gene due to its constitutive high expression in most cells [37]. In addition, we chose *pepsinogen C* (also known as progastricsin) as a reference gene in the stomach [38]. We evaluated the gene expression levels of the chitinases in a range of normal crab-eating monkey tissues (Figure 1A).

We designed several sets of primers for qPCR and evaluated their suitability based on a single Tm. We next constructed a standard DNA for qPCR by combining the four target fragments in a one-to-one ratio (Figure 1B,C). The 542 nucleotide-long standard DNA consisted of four cDNA fragments that covered the PCR target region (Figure 1B,C).

### 3.2. Expression of AMCase and CHIT1 in Normal Crab-Eating Monkey Tissues

**Figure 2:**
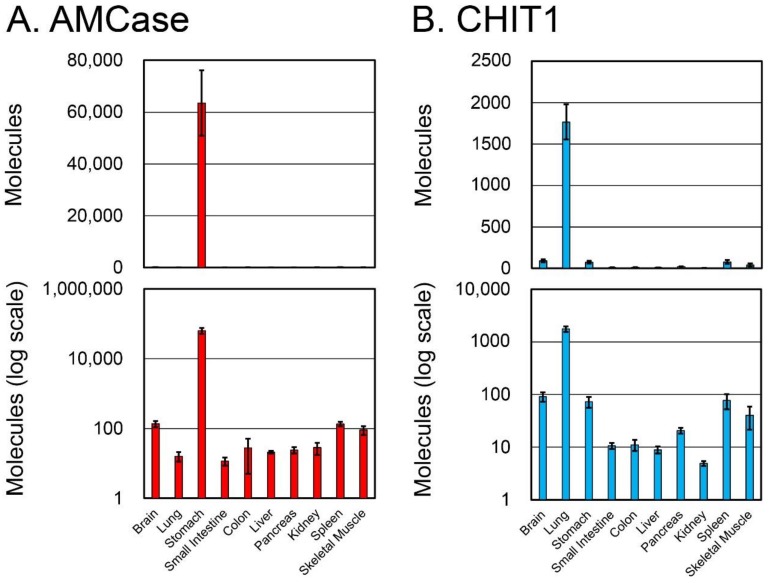
Expression of AMCase and CHIT1 messenger RNAs (mRNAs) in normal crab-eating monkey tissues. Quantification of (**A**) AMCase and (**B**) CHIT1 mRNA in crab-eating monkey tissues. Both chitinases were quantified by real-time PCR using the crab-eating monkey standard DNA. All values are expressed as number of molecules per 10 ng of total RNA in the *y*-axis. Upper panel, actual value; lower panel, logarithmic scale.

The total RNA from various normal crab-eating monkey tissues was analyzed using a qPCR assay with the specifically designed standard DNA (Figure 1). The resulting values were expressed as molecules per 10 ng of total RNA (Figure 2).

The highest levels of AMCase mRNA were detected in stomach, followed by brain, spleen, and skeletal muscle (Figure 2A). In contrast, CHIT1 mRNA was predominantly expressed in the lung, followed by brain, stomach, spleen, and skeletal muscle (Figure 2B). In other tissues, both the AMCase and CHIT1 mRNAs were expressed at relatively low levels.

### 3.3. Expression of AMCase, CHIT1, Pepsinogen C, and GAPDH mRNAs in Normal Crab-Eating Monkey Stomach and Lung Tissues

As described above, AMCase was predominantly expressed in the stomach, whereas CHIT1 mRNA was highly expressed in the lung (Figure 2). Importantly, many pathophysiological studies related to mammalian chitinases have been performed using stomach and lung. Thus, we next compared the expression levels of the chitinases and the reference genes (*pepsinogen C* and *GAPDH*) in these tissues.

**Figure 3:**
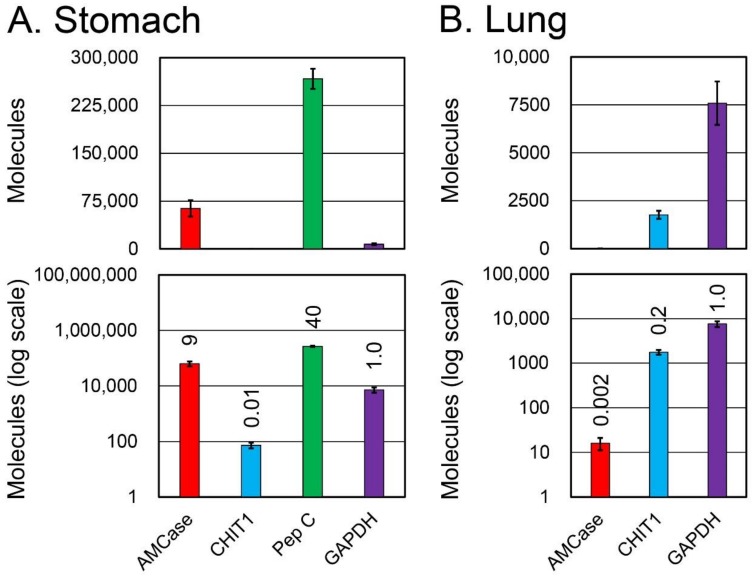
Analysis of AMCase and CHIT1 mRNAs and reference genes mRNA in stomach and lung tissues. Expression levels of the four genes determined using cDNAs prepared from normal crab-eating monkey (**A**) stomach and (**B**) lung were quantified by qPCR. The GAPDH expression level was set to 1.0; the values on the bars indicate the relative expression levels compared with that of GAPDH. Upper panel, actual value; lower panel, logarithmic scale.

When the GAPDH level was set to 1.0, the relative expression levels of AMCase, CHIT1, and pepsinogen C in normal monkey stomach tissue were 9, 0.01, and 40, respectively (Figure 3A). The AMCase mRNA levels were higher than those of GAPDH and lower than those of pepsinogen C in the monkey stomach. In the lung, on the other hand, the relative expression levels of AMCase and CHIT1 mRNAs were 0.002 and 0.2, respectively (Figure 3B). CHIT1 levels were lower than those of GAPDH while being 100 times higher than AMCase, which is otherwise higher in most monkey tissues. These results showed high relative expression levels of AMCase and CHIT1 in the stomach and lung, respectively.

### 3.4. Characterization of the qPCR System for Chitinase mRNA Level Comparison in Three Species Using a Monkey–Mouse–Human Hybrid DNA

**Figure 4:**
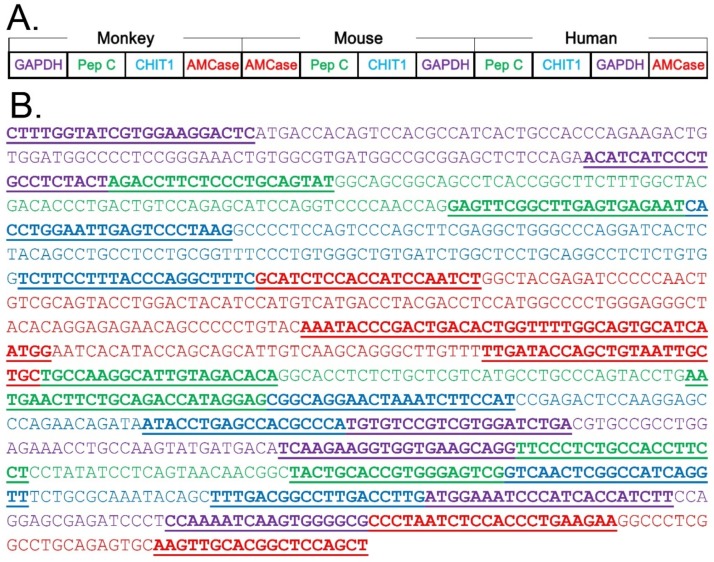
Strategy for comparing AMCase and CHIT1 mRNA levels between mouse, monkey, and human tissues. (**A**) Schematic representation of the monkey–mouse–human hybrid standard DNA used for the quantification. The target fragments for monkey, mouse, and human genes cDNAs were combined at a one-to-one ratio into a DNA fragment and used for the analysis of monkey, mouse, and human genes. (**B**) Nucleotide sequence of the standard hybrid DNA. The DNA contained twelve cDNA fragments (shown in different colors) covering the PCR target regions.

We next focused on the chitinase expression level comparison between mouse, crab-eating monkey, and human on a single scale using a specific qPCR system (Figure 4A). Mouse is the most frequently used animal model. We therefore constructed a hybrid standard DNA for qPCR by combining mouse, crab-eating monkey, and human standard DNAs (Figure 4B). The resulting 1086 nucleotide-long DNA contained twelve cDNA fragments that covered the PCR target regions for tested genes from all three species (see details in Figure 4B).

### 3.5. Comparison of Chitinase and Reference Gene mRNA Levels between Normal Mouse, Monkey, and Human Stomach Tissues

**Figure 5:**
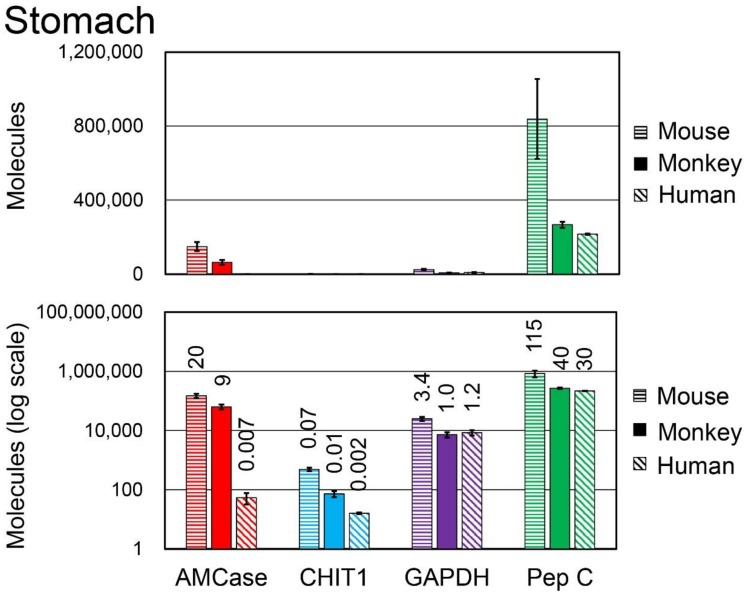
Analysis of the chitinase expression levels in mouse, monkey, and human stomach. The expression levels of *AMCase*, *CHIT1*, *GAPDH*, and *Pep C* were quantified using real-time PCR. Horizontal striped bars, mouse; filled bars, monkey; hatched bars, human. The expression level of the monkey *GAPDH* gene was set to 1.0; the values above the bars indicate the relative expression levels compared with that of monkey *GAPDH*. Upper panel, actual value; lower panel, logarithmic scale.

Next, we carried out cross-species gene expression analysis to compare normal mouse, monkey, and human total stomach RNA (Figure 5) using the monkey–mouse–human hybrid standard DNA (Figure 4).

The highest expression levels of AMCase and Chit1 mRNA were observed in mouse, followed by monkey and human. There was no significant difference in GAPDH mRNA expression between the species. As for pepsinogen C, expression in mouse was the highest, followed by monkey and human. When the GAPDH mRNA level was set to 1.0, the relative expression levels of mouse AMCase, monkey AMCase, human AMCase, mouse Chit1, monkey CHIT1, and human CHIT1 mRNAs were 20, 9, 0.007, 0.07, 0.01, and 0.002, respectively (Figure 5). The expression of AMCase in monkey and mouse was remarkably higher than in human.

### 3.6. Comparison of Chitinase and Reference Gene mRNA Levels between Normal Mouse, Monkey, and Human Lung Tissues

**Figure 6:**
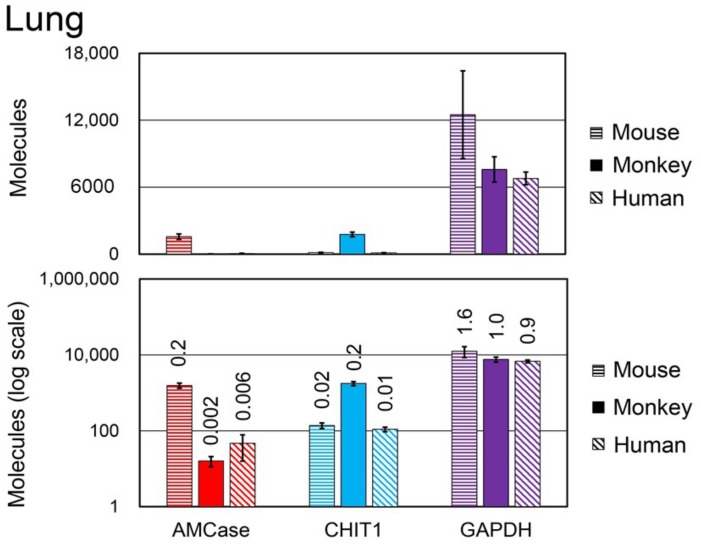
Analysis of the expression levels in monkey, human, and mouse lung tissues. The expression levels of *AMCase, CHIT1*, and *GAPDH* were quantified using qPCR. Horizontal striped bars, mouse; filled bars, monkey; hatched bars, human. The expression level of the monkey *GAPDH* gene was set to 1.0; the values above the bars indicate the relative expression levels compared with that of monkey *GAPDH*. Upper panel, actual value; lower panel, logarithmic scale.

Quantitative data obtained from lung tissues are shown in Figure 6. The relative expression levels of mouse AMCase, monkey AMCase, human AMCase, mouse Chit1, monkey CHIT1, and human CHIT1 mRNAs (GAPDH mRNA set to 1.0), were 0.2, 0.002, 0.006, 0.02, 0.2, and 0.01, respectively (Figure 6). The level of AMCase mRNA was the highest in mouse, followed by human and monkey. The expression of CHIT1 in monkey lung was higher than in human and mouse lungs.

### 3.7. Chitinase Levels and Activity in Mouse, Monkey, and Human Stomach and Lung

Next, we analyzed the enzymatic activity of the chitinases in the stomach or lung tissue extracts as the protein expression level using the synthetic substrate 4-methylumbelliferyl β-d-*N, N*′-diacetylchitobiose (4-MU-chitobiose). This substrate can be cleaved by both AMCase and CHIT1 while releasing the fluorogenic 4-MU molecules.

The pH profile for the chitinolytic activity differs between AMCase and CHIT1. Mouse AMCase shows a dual pH optimum with the major activity peak at around pH 2.0 followed by another peak at around pH 5.0 [8,27], whereas human AMCase has a broad optimal pH in the range of 2.0~5.0 [22,39]. The pH optimum for both mouse and human CHIT1 is relatively broad with the highest activity at around pH 5.0~6.0. In contrast to CHIT1, AMCase remains active at pH 2.0; therefore, this pH condition can be used to distinguish the chitinolytic activities of these two enzymes in the tissue extracts.

**Figure 7:**
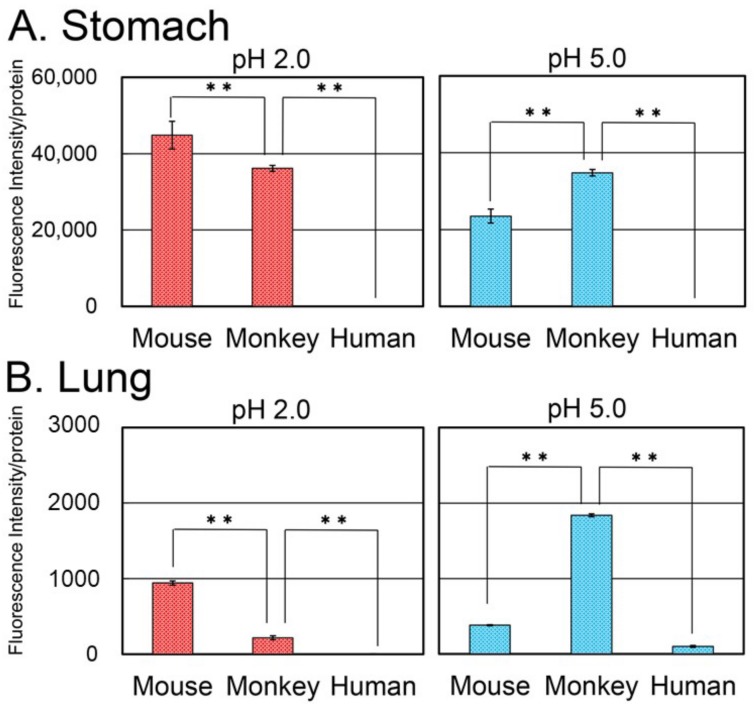
Chitinolytic activity in mouse, monkey, and human (**A**) stomach and (**B**) lung tissues. The activity was measured using a fluorogenic chitin substrate, 4-MU-chitobioside. Chitinolytic activity at pH 2.0 or pH 5.0 is expressed on the *y*-axis: red bars, pH 2.0; blue bars, pH 5.0. Fluorescence intensity is shown as activity per 16 µg protein. ** *p*  <  0.01.

At pH 2.0, we detected robust chitinolytic activity in mouse and monkey stomach extracts, whereas only low activity was observed in the human tissue (Figure 7A). These activities can be attributed to AMCases because, as mentioned above, CHIT1 is inactive at pH 2.0. At pH 5.0, the chitinolytic activity in the monkey stomach extract was higher than in the mouse extract. Since monkey AMCase has been shown to have highest activity at pH 5.0 [34], this data is essentially consistent with the AMCase mRNA levels in stomach tissues (Figure 5).

As for the lung tissues, chitinolytic activity was high in mouse followed by monkey at pH 2.0, while it was undetectable in the human lung. Similar to the result from stomach tissues (see above), AMCase is responsible for the activity at these conditions. As anticipated, chitinolytic activity at pH 5.0 was higher than that at pH 2.0 in monkey lung and even exceeded that of human lung extract at pH 2.0 (Figure 7B). These results show that the levels of CHIT1 activity are consistent with the mRNA levels in lung tissues (Figure 6).

## 4. Discussion

The crab-eating monkey is an important nonhuman primate model for biomedical research and drug development [31]. However, detailed gene expression and biochemical analysis of the monkey chitinases has not been performed so far. Gene expression analysis followed by biochemical enzyme assays are crucial steps to gain insight into the in vivo regulation of mammalian chitinases. In this report, we investigated differences between the crab-eating monkey and human in the mRNA and protein levels. The presented data indicate that expression of AMCase and CHIT1 is altered between monkey and human, although they are genetically and physiologically similar.

The conventional real-time reverse transcriptase-coupled PCR (RT-PCR) system is widely used due to its simplicity. However, there are limitations in quantifying multiple mRNAs in a single specimen. We established a qPCR system allowing absolute quantification of the number of different genes using the same scale [25,26,27]. This method enables a direct comparison of gene expression levels among target genes.

AMCase mRNA was predominantly expressed in crab-eating monkey stomach. It is expressed at a level higher than that of GAPDH and comparable to that of pepsinogen C, a major component of the gastric mucosa. In monkey lung, on the other hand, CHIT1 is present at levels close to that of GAPDH and 100 times higher than that of AMCase, whose expression otherwise overtops that of CHIT1 in most other monkey tissues. Thus, AMCase and CHIT1 are major transcripts in the monkey stomach and lung tissues, respectively.

We performed a cross-species gene expression analysis in mouse, monkey, and human using qPCR. We found that mouse and monkey stomach expressed a large amount of AMCase, whereas the amount was very low in human. We also measured the chitinolytic activity of the enzymes in the stomach or lung tissue extracts and observed robust activities in the mouse and monkey stomach tissues, but not in human, in accordance with the mRNA expression levels.

Mouse and pig AMCase, as well as chicken Chia, have been reported to function as major protease-resistant glycosidases in the stomach and intestine [27,28,29]. Studies have shown that some insect-eating monkeys underwent a similar physiological adaptation as those observed in mouse, chicken, and pig as well as in insectivorous bats [27,28,29,40,41]. Monkey and mouse are omnivorous animals with chitin-containing organisms included in their diet. For example, in contrast to modern humans, crab-eating monkeys consume whole crabs and insects.

Robust chitinolytic activities were observed in both mouse and monkey stomachs, but not in human. Since monkey AMCase is most active at around pH 5.0 [34], the activity of the monkey stomach extract was higher than that of the mouse extract at pH 5.0. The pH in the monkey stomach shifts around neutral range between pH 5.0 and pH 7.0 after feeding [42]. Therefore, monkey AMCase may work as a digestive enzyme in the stomach, similarly to that of mouse and pig and Chia of chicken [27,28,29]. Accordingly, the chitinolytic activity of human stomach was significantly lower than those of mouse and monkey stomach at pH 2.0. Thus, feeding behavior affects AMCase expression levels and determines chitin digestibility in the particular organisms [30].

AMCase has been shown to play a role in defense against environment-derived chitin accumulating in the respiratory tract, and regulation of the expression of profibrotic cytokines [23]. Our present study showed that in lungs, AMCase mRNA in monkey and human is similar, while in mouse, the AMCase mRNA level is significantly higher compared with both primates. We detected the highest chitinolytic activity in the monkey lung extract, followed by mouse and human. Therefore, monkey can be a more useful animal model than mouse in research related to lung pathologies, including asthma, as AMCase has been known to be related to this disease [19,20,21,22,23,24]. However, the 50 times higher activity of the monkey AMCase as compared to human [34] should always be taken into account.

Based on our results, we assume that crab-eating monkeys have a potent biodefence system in lungs probably also due to their habitat with substantial amounts of airborne fungi. The expression profiles of CHIT1 in the lungs of the tested species differ from those of AMCase. As for the monkey, the CHIT1 mRNA level is significantly higher than that of AMCase. It is also markedly higher than in mouse and human. In a previous report, we have shown that the expression levels of CHIT1 in lung are conserved between mouse and human [26]. Interestingly, therapeutic administration of exogenous Chit1 to the airways restored the ability to clear or degrade naturally acquired chitin in AMCase-deficient mice [23]. Thus, we assume that CHIT1 in monkey lung acts as biological defense against chitin-containing organisms such as inhaled fungi in a similar way as AMCase in the mouse lung. Further research on the chitinase activity of monkey CHIT1 is needed to confirm this function of the enzyme in detail.

From the evolutionary point of view, the crab-eating monkey is located between mouse and human. Thus, the crab-eating monkey is often preferred over mouse as a model animal more plausibly recapitulating human conditions in fields of pharmacology and pathology. In this study, we provide three important results. Firstly, expression of chitinase genes is tissue-specific in monkey as well as in mouse and human. Secondly, the gene expression pattern of AMCase and CHIT1 varies between normal mouse, monkey, and human. Finally, these mRNA differences among mouse, monkey, and human stomach tissues reflect chitinolytic activity differences. These data are important for choosing an animal model for specific experiments. Our present results will also provide hints towards understanding the biological function of chitinases, especially in pathophysiological studies using animal models and related to human diseases. Elucidating the functions of the chitinases in the crab-eating monkey is needed for drug development as well as for pathophysiological research.

## 5. Conclusions

We performed gene expression analysis of chitinases in normal crab-eating monkey tissues using a qPCR system and using a single standard DNA. AMCase mRNA was expressed at an exceptionally high level in the stomach, whereas CHIT1 mRNA was highly expressed in the lung. In addition, we compared AMCase and CHIT1 mRNA levels between mouse, monkey, and human. Gene expression of AMCase and CHIT1 varies between tested species. Moreover, these mRNA differences among the species were essentially reflecting differences in the chitinolytic activities. These data provide gene expression information in monkeys and will help in biomedical research using the crab-eating monkey.

## Supplementary Materials

The following are available online at http://www.mdpi.com/2073-4425/9/5/244/s1. Table S1. List of qPCR primers.
