# Supplementary material for: Chitinase mRNA Levels Determined by QPCR in Crab-Eating Monkey (Macaca fascicularis) Tissues: Species-Specific Expression of Acidic Mammalian Chitinase and Chitotriosidase
Source: Genes (Basel). 2018 May 9;9(5):244. doi: 10.3390/genes9050244 (PMC5977184; doi:10.3390/genes9050244)
Supplement: Supplementary file 1 [file genes-09-00244-s001.pdf]

Table S1. List of qPCR primers.

Gene	Forward primer	Reverse primer
Monkey AMCase	GCATCTCCACCATCCAATCT	CCAGTGTCAGTCGGGTATTT
Monkey CHIT1	CACCTGGAATTGAGTCCCTAAG	GAAAGCCTGGGTAAAGGAAGA
Monkey Pepsinogen C	AGACCTTCTCCCTGCAGTAT	ATTCTCACTCAAGCCGAACTC
Monkey GAPDH	CTTTGGTATCGTGGAAGGACTC	AGTAGAGGCAGGGATGATGT
Mouse AMCase	TTTTGGCAGTGCATCAATGG	GCAGCAATTACAGCTGGTATCAA
Mouse Chit1	CGGCAGGAACTAAATCTTCCAT	TGGGCGTGGCTCAGGTAT
Mouse Pepsinogen C	TGCCAAGGCATTGTAGACACA	CTCCTATGGTCTGCAGAAGTTCATT
Mouse GAPDH	TGTGTCCGTCGTGGATCTGA	CCTGCTTCACCACCTTCTTGA
Human AMCase	CCCTAATCTCCACCCTGAAGAA	AGCTGGAGCCGTGCAACTT
Human CHIT1	GTCAACTCGGCCATCAGGTT	CAAGGTCAAGGCCGTCAAA
Human Pepsinogen C	TTCCCTCTGCCACCTTCCT	CGACTCCCACGGTGCAGTA
Human GAPDH	ATGGAAATCCCATCACCATCTT	CGCCCCACTTGATTTTGG
